# Multi-Modal Vehicle Trajectory Prediction by Collaborative Learning of Lane Orientation, Vehicle Interaction, and Intention

**DOI:** 10.3390/s22114295

**Published:** 2022-06-05

**Authors:** Wei Tian, Songtao Wang, Zehan Wang, Mingzhi Wu, Sihong Zhou, Xin Bi

**Affiliations:** 1 School of Automotive Studies, Tongji University, Shanghai 201804, China; tian_wei@tongji.edu.cn (W.T.); wangsongtao@tongji.edu.cn (S.W.); wangzehan@tongji.edu.cn (Z.W.); zhousihong@tongji.edu.cn (S.Z.); 2 Nanchang Automotive Institute of Intelligence & New Energy, Nanchang 330044, China; wumingzhi@naiine.com

**Keywords:** trajectory prediction, lane coordinate transform, intention learning, multi-modal trajectory

## Abstract

Accurate trajectory prediction is an essential task in automated driving, which is achieved by sensing and analyzing the behavior of surrounding vehicles. Although plenty of research works have been invested in this field, it is still a challenging subject due to the environment’s complexity and the driving intention uncertainty. In this paper, we propose a joint learning architecture to incorporate the lane orientation, vehicle interaction, and driving intention in vehicle trajectory forecasting. This work employs a coordinate transform to encode the vehicle trajectory with lane orientation information, which is further incorporated into various interaction models to explore the mutual trajectory relations. Extracted features are applied in a dual-level stochastic choice learning to distinguish the trajectory modality at both the intention and motion levels. By collaborative learning of lane orientation, interaction, and intention, our approach can be applied to both highway and urban scenes. Experiments on the NGSIM, HighD, and Argoverse datasets demonstrate that the proposed method achieves a significant improvement in prediction accuracy compared with the baseline.

## 1. Introduction

In recent years, automated driving has become an emerging research area in applied intelligence. For an automated driving vehicle, the correct motion planning and behavior decision not only rely on the perceived positions of surrounding objects (especially cars), but also on their future trajectories [1]. The latter information, however, can only be provided by trajectory prediction approaches. Despite the tremendous progress in related studies [2,3,4,5], vehicle trajectory prediction is still a challenging task, mainly due to the environment’s complexity and the driving intention uncertainty.

To address the complexity of the driving environment, it is necessary to comprehensively consider various kinds of information to accurately predict the vehicle trajectory. Recent research works have adopted the dynamic relation, i.e., the interaction, between the target car and its surrounding vehicles. Typical methods are social pooling [2], convolutional social pooling [3] and the pooling module [4]. Although these models have been validated with good accuracy, the temporal dependence of the interaction on the trajectory states is still unexplored. Since each observed frame plays a different role in the trajectory prediction, the question of how to efficiently embed the interaction into the learning procedure still remains open. For traffic participants that travel along the road, their motion changes with the road structure. Thus, another method is to deploy road information. Related works can be categorized into two paradigms: rasterization and vectorization [2,6]. However, these algorithms require much road map processing, which is disadvantageous for lightweight implementations. As the driver intention significantly affects the vehicle trajectory and is hard to anticipate, multi-modal trajectory prediction has become one of the trends in current research. These algorithms either define all motion modes in advance [3] or iteratively sample latent variables for multi-modal trajectory generation, which easily results in mode collapse [4].

To address the above-mentioned problems, this paper proposes a collaborative learning architecture for vehicle trajectory prediction (codes will be available at https://github.com/tongji-wangsongtao/vehicle-trajectory-prediction-by-collaborative-learning, accessed on 4 June 2022). Our contributions are summarized as follows:

- We explore the embedding efficiency of various interaction models under the Long Short-Term Memory (LSTM) encoder–decoder prediction framework and integrate both interaction and lane orientation information in a lightweight encoding module to improve environment interpretation.
- We propose an approach to effectively learn the driving uncertainty, which conforms to the lane orientation and considers multiple modes of the vehicle trajectory in a dual-level mode paradigm.
- We perform extensive validation on both high-speed scenarios and urban road scenarios, including the NGSIM, HighD, and Argoverse benchmarks. Experimental results show that the proposed method significantly improves the prediction accuracy compared with the baselines while maintaining a fast runtime speed.

## 2. Related Work

The trajectory prediction task in autonomous driving aims to predict the future motion state of surrounding objects, especially cars. In this section, the recent research progress in this field is introduced regarding three aspects most relevant to this paper, as follows.

### 2.1. Trajectory Prediction Based on the Interaction Model

Accurate vehicle trajectory prediction should consider the dynamic relation between the target car and its surrounding vehicles. At present, interaction modeling in trajectory prediction can be roughly divided into two categories: interaction through a spatial grid [2,3,7] and interaction based on an attention mechanism [4,8,9,10]. In the first approach, the spatial grid is centered on the predicted target. Historical trajectories of both the target car and its surrounding vehicles are encoded and assigned to the corresponding spatial grids according to their positions. The grid-aligned features are formed into a tensor, as in social pooling, from which the mutual spatial relationship between vehicles can be learned by Neural Networks (NNs) to predict the future trajectory [2]. Different from the first approach, the attention-based method explicitly considers the interaction between the target car and its surrounding vehicles in a pairwise manner. In the work on the pooling module [4], relative spatial distance was used as the weight of the influence of the surrounding vehicle on the predicted target. Zhang et al. [9] also proposed an interaction model that selectively considers the important information from surrounding traffic participants. Since the weight has to be calculated for each individual interaction, their computation load also increased. Furthermore, the road-sharing scenario between human-driven and automated vehicles was well studied by Saeed et al. [11,12].

### 2.2. Trajectory Prediction Based on Road Map Information

Road map information has been widely used in vehicle trajectory prediction approaches and has demonstrated improved performance. Since High-Definition (HD) maps have become a common tool used in autonomous driving systems, the utilization of road map information for trajectory prediction is in line with practical applications. Generally, these works can be divided into two categories: raster-image-based encoding and road map vectorization. In the first category, the road map information with a top-view perspective is directly rendered as an RGB image, which favors image processing approaches. For instance, Zhang et al. [7] applied Convolutional Neural Networks (CNNs) to extract scene feature vectors from the road map image. Park et al. [13] proposed a semantic raster map, with each color channel corresponding to one of the specific scene elements such as the lane, drivable area, and lane centerline. The Uber research team [14,15,16,17] proposed new CNNs to fuse the historical vehicle trajectory on the basis of the semantic grid map and further improved the vehicle trajectory prediction. Another way to consider the road map is road map vectorization. Compared with image-based encoding, this kind of approach focuses more on the topology information of a traffic scene. Luo et al. [18] interpreted the lane centerline as a feature vector, which was fed into the network in conjunction with an additional attention mechanism. Gao et al. [6] represented various traffic elements such as lane centerlines and parking lines as graph nodes and employed a Graph Neural Network (GNN) to predict future vehicle trajectories. Greer et al. [19] designed an auxiliary loss to address failure cases due to the off-road rate by penalizing trajectories that oppose the heading direction (flow direction) of a driving lane.

### 2.3. Multi-Modal Trajectory Prediction

Due to the uncertainty of driving intention, multi-modal prediction is necessary. However, multi-modal trajectory prediction is an essential, but challenging task. According to whether the modes are pre-defined, current approaches can be divided into the following two categories. In the first type, all the motion modes of the predicted target are defined in advance. The network predicts multi-modal trajectories according to pre-defined mode codes at the input side. For instance, Deo et al. [3] divided the motion modes of vehicles in a high-speed scene into six independent categories. Each mode was fed into the prediction network in parallel in the form of one-hot encoding. The vehicle motion in a high-speed scene was further divided by Hu et al. [20] into three modes: straight ahead, cutting in, and cutting out. The above methods need to annotate the modes of the training data in advance. On the contrary, the second type directly learns the multi-modality of target motion by utilizing latent variables to represent the mode distribution, e.g., by a Monte Carlo sampling process. Gupta et al. [4] applied latent variables to the network learning similar to the Generative Adversarial Network (GAN) to generate more reasonable trajectories. Huang et al. [21] designed an efficient sampling method of latent variables to cover the possible future actions and their likelihoods. Although the above methods do not require modality annotations for the utilized dataset, the trajectory distribution obtained by the sampling method has difficulty covering all possible motion modes, which may lead to mode collapse.

**Figure 1:**
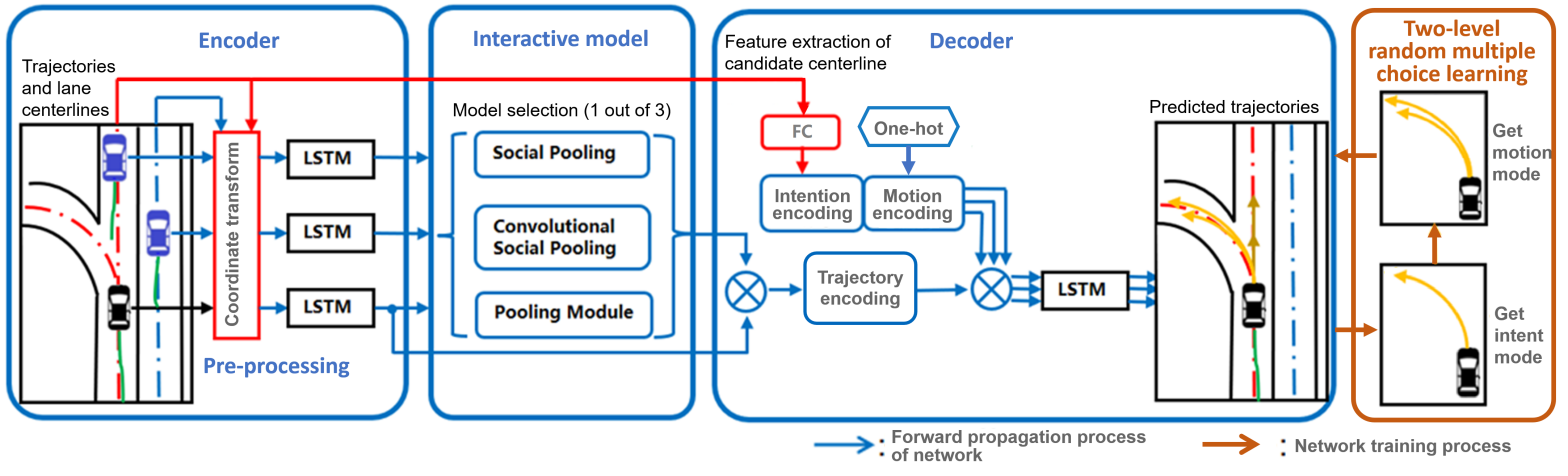
The multi-modal vehicle trajectory prediction framework based on interaction modeling and lane orientation information. Related lane centerline is depicted in red. Predicted trajectories are represented in yellow, while historical trajectories are in green.

Based on the prior work [22,23], the proposed approach in this paper adopts random multiple choice learning to optimize the multi-modal trajectory prediction network and implicitly decouples the vehicle motion modes into two levels. Moreover, to avoid the heavy computation by processing road maps, this paper focuses on the lane orientation information, which is encoded by a lightweight auxiliary module based on a coordinate transform. Furthermore, efficient embedding of various interaction models into the LSTM encoder–decoder architecture is explored to improve the accuracy of predicted trajectories, as shown in Figure 1. By a random multi-choice learning of the lane orientation and vehicle interaction and intention, the effectiveness of the proposed approach is verified by both qualitative and quantitative experiments in highway and urban scenarios.

## 3. Multi-Modal Trajectory Prediction by Joint Learning Interaction and Lane Orientation Information

Given the historical positions of a target vehicle *O*, which can be achieved by on-board perception and localization systems, its historical trajectory can be denoted as $X_{o}=\left[\left(x_{o}^{t-k_{o}+1},y_{o}^{t-k_{o}+1}\right),\ldots ,\left(x_{o}^{t-1},y_{o}^{t-1}\right),\left(x_{o}^{t},y_{o}^{t}\right)\right]$, with $X_{o}^{t}=\left(x_{o}^{t},y_{o}^{t}\right)$ indicating the position coordinates at time step *t*. $k_{o}$ is the number of historical positions. The future trajectory of target *O* in the next $l_{o}$ time steps is denoted as $Y_{O}=\left[\left(x^{t+1},y^{t+1}\right),\ldots ,\left(x^{t+l_{o}},y^{t+l_{o}}\right)\right].$

Since the trajectory prediction can be regarded as a sequence generation problem, the LSTM encoder–decoder architecture [24] is adopted as the backbone prediction architecture in this paper. In the encoding phase, the historical position is firstly processed by a fully connected layer (FC, with 32 neural units) to extract features, which are further passed to an LSTM to generate trajectory encodings. Mathematically, the encoding stage can be interpreted as

(1) $$\left\{\begin{array}{cc} e^{t} & ={\mathrm{FC}}_{e}\left(X_{o}^{t}\right)\\ g_{e}^{t} & =e^{t}\\ \left(h_{e}^{t},m_{e}^{t}\right) & ={\mathrm{LSTM}}_{e}\left(h_{e}^{t-1},m_{e}^{t-1},g_{e}^{t}\right) \end{array}\right.,$$

where $g_{e}^{t},e^{t}$ are intermediate variables. $\left(h_{e}^{t},m_{e}^{t}\right)$, respectively, indicate the hidden state and the cell state of the LSTM encoder with zero initialization. At the decoding stage, another LSTM is utilized, whose hidden state is processed by a subsequent FC layer with the same structure to yield the predicted position at the next time step, interpreted as

(2) $$\left\{\begin{array}{cc} d^{t} & ={\mathrm{FC}}_{e}\left(Y_{o}^{t}\right)\\ p_{d}^{t} & =d^{t}\\ \left(h_{d}^{t+1},m_{d}^{t+1}\right) & ={\mathrm{LSTM}}_{e}\left(h_{d}^{t},m_{d}^{t},p_{d}^{t}\right)\\ Y_{o}^{t+1} & ={\mathrm{FC}}_{d}\left(h_{d}^{t+1}\right) \end{array}\right.,$$

where $p_{d}^{t},d^{t}$ are intermediate variables. $\left(h_{d}^{t},m_{d}^{t}\right)$, respectively, indicate the hidden state and the cell state of the LSTM decoder and are initialized as $\left(0,m_{e}^{n}\right)$.

To model the interaction between vehicles, the trajectories of the target *O* and all its *L* neighbors should be considered. Therefore, the inputs can be expressed as a tensor: $X=\{X_{O},X_{1},X_{2},\ldots ,X_{L}\}.$ The tensor X is firstly processed by the LSTM encoder, whose state H is passed to the interaction model $\phi_{r}\left(\cdot \right)$ to extract the interaction feature, interpreted as

(3) $$r_{int}^{t}=\phi_{r}\left(H^{t}\right),$$

where $H^{t}$ indicates the LSTM state at time *t*, while $r_{int}^{t}$ corresponds to the interaction feature vector.

### 3.1. Embedding of Interaction Model

**Figure 2:**
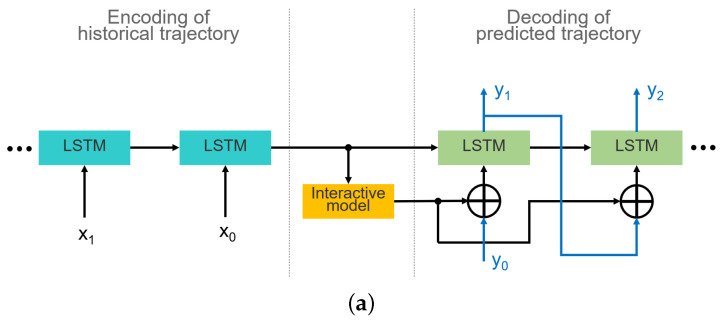

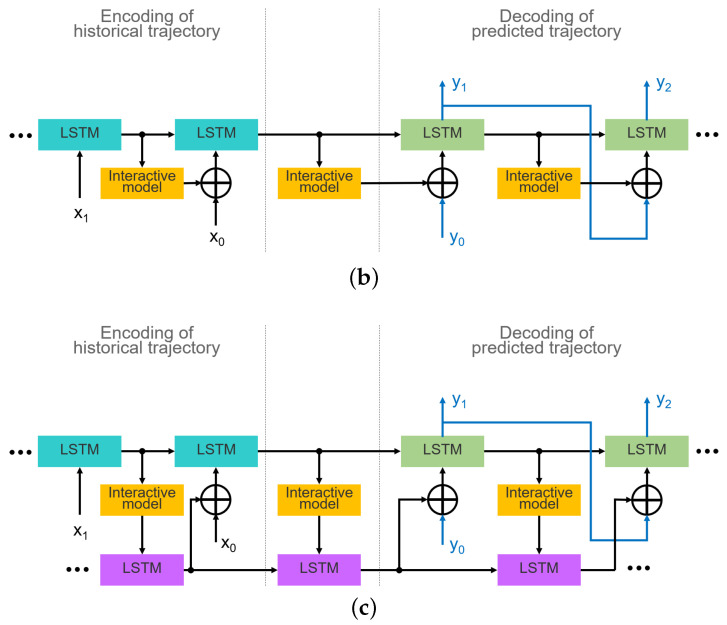
Three interaction model embedding methods in the LSTM encoder–decoder network. (**a**) Embedding interaction model only at the current frame. (**b**) Embedding interaction model at each frame. (**c**) Embedding interactive model with spatial–temporal coupling.

There are two aspects to be considered when modeling the interaction relationship in a prediction network: the interaction model and its embedding manner. Since a brief introduction about various interaction models is presented in Section 2, here, we explore three embedding methods: embedding only at the current frame, embedding at each frame, and embedding with spatial–temporal coupling, as shown in Figure 2. The first embedding implies that the interaction is preserved until the current frame. Thus, the input of the LSTM decoder in Equation (2) is modified as

(4) $$p_{d}^{t}=d^{t}⊕r_{int}^{0},$$

where $r_{int}^{0}$ denotes the interaction feature at current time t=0 and ⊕ indicates the concatenation operation. The second one assumes that the interaction constantly changes and thus should be reconsidered at every moment. The modification in the LSTM encoder is

(5) $$g_{e}^{\tau }=e^{\tau }⊕r_{int}^{\tau }$$

and in the LSTM decoder is

(6) $$p_{d}^{t}=d^{t}⊕r_{int}^{t},$$

where τ,t, respectively, indicate the time point of the encoding and decoding process. The third embedding also assumes a constantly changing interaction, which is correlated between frames. The modification in the LSTM encoder is

(7) $$\left\{\begin{array}{cc} \left(h_{r}^{\tau },m_{r}^{\tau }\right) & ={\mathrm{LSTM}}_{r}\left(h_{r}^{\tau -1},m_{r}^{\tau -1},r_{int}^{\tau }\right)\\ g_{e}^{\tau } & =e^{\tau }⊕h_{r}^{\tau } \end{array}\right.$$

and in the LSTM decoder is

(8) $$\left\{\begin{array}{cc} \left(h_{r}^{t},m_{r}^{t}\right) & ={\mathrm{LSTM}}_{r}\left(h_{r}^{t-1},m_{r}^{t-1},r_{int}^{t}\right)\\ p_{d}^{t} & =d^{t}⊕h_{r}^{t} \end{array}\right.,$$

where the frame correlation is interpreted by an additional network ${\mathrm{LSTM}}_{r}$. Its hidden state and cell state $\left(h_{r}^{t},m_{r}^{t}\right)$ are initialized at zero.

To evaluate the three above embedding methods, comparison studies are conducted in Section 4. Based on the experimental results, we empirically selected the first embedding method in our framework due to a trade-off between the prediction accuracy and time complexity.

### 3.2. Integration of Lane Orientation Information

As the road structure is a fundamental component of the road scene and significantly affects the driving status of vehicles, considering road map information within the network could improve both the prediction accuracy and the generalization ability to various traffic scenarios. Since on-road traversing vehicles should adhere to their corresponding lanes, the lane centerline is an essential part of the road information, especially the orientation. In this paper, the lane centerline information is used to represent the lane orientation and integrated in the prediction framework by transforming the vehicle trajectory from the world coordinate system to the lane centerline coordinate system, which consists of the following two steps.

#### 3.2.1. Selection of Candidate Lane Centerline

Given two points *A* and *B* on the road map, their distance is measured by the Manhattan distance, expressed as

(9) $$s_{M}=|x_{A}-x_{B}\left|+\right|y_{A}-y_{B}|,$$

where $\left(x_{A},y_{A}\right)$ and $\left(x_{B},y_{B}\right)$ denote the world coordinates of point *A* and point *B* on the road map. The candidate lane centerlines are selected by a Breadth-First Search (BFS). The lower threshold of the Manhattan distance $\theta_{M}$ is firstly defined. Then, lane centerlines that are within the threshold $\theta_{M}$ from the predicted vehicle position are extracted from the road map. If no lane centerlines are found intersecting with the searching area, the searching distance, i.e., $\theta_{M}$, is increased. The process is repeated until a lane centerline is found.

#### 3.2.2. Motion Trajectory Coordinate Transform

**Figure 3:**
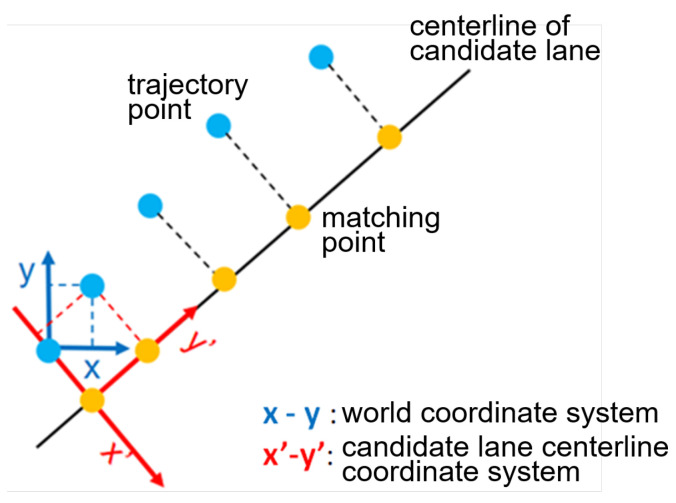
Coordinate transform of historical vehicle trajectory.

Given the candidate lane centerline that the target vehicle may traverse in the future, its historical trajectories should be mapped from the world coordinate system to the candidate lane centerline coordinate system, as shown in Figure 3. Here, each trajectory point is firstly projected onto the candidate lane centerline. The coordinate system of the candidate lane centerline is defined with its *y*-axis along the lane direction, while the *x*-axis is in the perpendicular direction and follows the right-hand principle. The origin point is defined as the projected point of the current vehicle position projected on the lane centerline.

In highway scenarios, the lane centerlines are mostly parallel to each other. Therefore, the integration of road map information can be further simplified by converting vehicle trajectories into the coordinate system of the same lane centerline, i.e., a lane-level conversion.

### 3.3. Multi-Modal Prediction

Generally, the trajectory modes of the target can be divided into two levels: the intention mode and the motion mode [23]. However, such a division is not applicable to urban scenes. In an urban scene, the vehicle trajectory modes rely on road structures. Since the road structure dynamically changes, it is difficult to define and generate all the vehicle modes in advance. Therefore, this paper exploits a multi-modal trajectory prediction adapted to the dynamic road structures. Inspired by the dual-level modality characteristics defined for the vehicle trajectory, this paper directly predicts the candidate lane centerline instead of the intention mode. The proposed approach also employs the second modality level, i.e., the motion mode, which is used to measure the speed change of a vehicle driving on a candidate lane. Therefore, the predicted vehicle trajectories still correspond to a combination of various intention and motion modes, yet the number of predicted trajectories changes dynamically with the road structures.

#### 3.3.1. Modality Interpretation

In the network, both the historical trajectory of the target and its interaction with surrounding vehicles are firstly transformed into lane centerline coordinates and fed into the LSTM encoder to generate trajectory encoding features. The trajectory encoding vector is further concatenated with its intention and motion mode encoding. As previously mentioned, the candidate lane centerlines correspond to intention modalities. Thus, one-hot encoding is inappropriate to encode the intention modes, as the code number should be fixed in advance. Moreover, the trajectory is predicted in the candidate lane centerline coordinates. With one-hot encoding, the network cannot learn the driving characteristics of a vehicle along different types of lane centerlines. Therefore, an additional FC layer is exploited to extract a feature vector from the candidate lane’s centerline to encode the corresponding intention mode, as shown in the decoder part in Figure 1.

To obtain the centerline feature vector, starting from the projected point of the current vehicle position, 30 points with an equal length are sampled from the candidate lane centerline. Thereafter, the coordinates of sampled points relative to the starting point are concatenated and flattened into a vector with a length of 60. This vector is further fed into the FC layer (with 32 neural units) to generate the intention encoding. As the number of motion modes is fixed, one-hot encoding is still used to represent different motion modalities. Thus, the input $p_{d}^{t}$ of the LSTM decoder can be expressed as

(10) $$\left\{\begin{array}{c} \nu_{line}\in \psi_{l}\left(M\right)\\ \gamma_{line}={\mathrm{FC}}_{l}\left(\nu_{line}\right)\\ \gamma_{m}\in \psi_{m}\left(N\right)\\ p_{d}^{t}=d^{t}⊕r_{int}⊕\gamma_{line}⊕\gamma_{m} \end{array}\right.,$$

where $\psi_{l}\left(M\right)$ is the set of *M* candidate lane centerlines; $v_{line}$ is the vector composed of 30 points sampled from the corresponding candidate lane centerline; $\gamma_{line}$ is the feature vector of the candidate lane centerline generated by the FC layer; $\gamma_{m}$ represents one of the *N* motion mode encodings (one-hot); $\psi_{m}\left(N\right)$ is the corresponding set.

#### 3.3.2. Learning Strategy

In addition, the multi-modal trajectory prediction network is trained with the improved random multiple-choice learning approach based on [20]. In such a training process, the key is to design a reasonable arbitration scheme to select the “winner” trajectory, which can well match the ground-truth from all predicted trajectories. The searching process of the optimal trajectory here is divided into two phases. In the first phase, the optimal intention mode is determined. On each candidate lane centerline, the matched points of the ground-truth trajectory are calculated. Matched point pairs with a Manhattan distance under a pre-defined threshold are selected. All candidate lane centerlines are sorted according to the number of selected point pairs. The one with the most point pairs is considered as the “winner” intention mode, denoted by $m^{*}$. In the second phase, based on the selected intention mode, i.e., along the corresponding candidate lane centerline, the “winner” motion mode is determined as the predicted trajectory with the minimal Average Displacement Error (ADE) with respect to the ground-truth and denoted as $n^{*}$. The selection process can be interpreted as

(11) $$\mathrm{ADE}\left(Y_{m^{*},n},\bar{Y}\right)=\frac{1}{l_{o}}\sum_{i=1}^{l_{o}}∥\left({\bar{x}}^{i},{\bar{y}}^{i}\right)-\left(x_{m^{*},n}^{i},y_{m^{*},n}^{i}\right)∥,$$

(12) $$n^{*}=\underset{n\in N}{\arg\min}L_{ADE}\left(Y_{m^{*},n},\bar{Y}\right),$$

where ground-truth values are marked by $\;\bar{}\;$.

As mentioned above, only the most promising trajectory proposal is regarded as the “correct mode” and selected during the training. In this way, the output of each mode is specialized on a subset of the training data, thus guaranteeing the learning of the multiple modalities of the trajectories. By selecting the winner trajectory, the regression loss is defined as $L_{\mathrm{ADE}}$ $\left(Y_{m^{*},n^{*}},\bar{Y}\right)$, which is a smooth L1 loss for ADE. Additionally, a classification head is added to the network, which is used to filter out impractical predictions at the inference stage. The label of the “winner” is set to 1, and the label of others is set to 0. The classification loss $L_{class}$ is in a cross-entropy form, and the prediction loss can be defined as

(13) $$L_{pred}=L_{class}+\alpha L_{\mathrm{ADE}}\left(Y_{m^{*},n^{*}},\bar{Y}\right),$$

where the hyper-parameter α is a trade-off between the two losses.

## 4. Experiments

### 4.1. Experimental Setting

For an extensive evaluation of the proposed prediction framework, three publicly available benchmarks, i.e., NGSIM [25], HighD [26], and Argoverse [27], were employed in the experiments. The NGSIM and HighD benchmarks are both constructed for highway scenarios. In the above two datasets, a 5 s future trajectory is predicted based on a 3 s historical trajectory of the target with a sampling rate of 5 Hz. Argoverse is an urban dataset providing lane-level maps. This dataset only provides the ground-truth trajectory of a vehicle within 5 s (with a sampling frequency of 10 Hz). The evaluation policy of this dataset requires using the historical trajectory in the first 2 s to predict the trajectory in the last 3 s. Although our approach is not limited by the prediction time, we still followed the evaluation policy. We adopted N=2 motion modes in learning, while the intention mode number *M* was equal to the lane candidate number in urban scenarios. Our proposed prediction architecture was implemented using PyTorch. The learning rate was set to 1 × 10 ${}^{-3}$ during training, and the Adam optimizer was adopted for optimization.

For the evaluation metrics, in addition to the ADE, the Final Differential Error (FDE) was chosen in this paper. For the multi-modal prediction network, we used the minADE and minFDE as additional evaluation criteria.

### 4.2. Exploration of Interaction Integration

As previously mentioned, two points should be considered during the integration of interaction information: the interaction model and its embedding method, both of which are explored as follows.

#### 4.2.1. Exploration of Interaction Model

To comprehensively analyze the influence of the interaction relationship on prediction accuracy, three interaction models were evaluated in the experiment. In this paper, social pooling [2] and convolutional social pooling [3] were selected as the representatives of interaction modeling through spatial grids, while the pooling module [4] was selected as the representative of interaction modeling based on the attention mechanism. The maximum number of surrounding vehicles considered in the interaction with the target was set to 5. To ease the experiment, as well as maintain the fairness of the comparison, all interaction models were embedded at the current frame in the single-mode vehicle trajectory prediction network, resulting in three network versions denoted as S-LSTM, CS-LSTM, and P-LSTM, respectively. Here, we output only one trajectory per target vehicle. The above three networks were compared with the baseline network of the LSTM encoder–decoder architecture without the integration of interaction information. Additionally, a Kalman filter [28] based on Constant Velocity (CV) and an Intelligent Driver Model (IDM) [29] were also compared in the experiment. The evaluation results are shown in Table 1.

It can be seen that on both the NGSIM and HighD datasets, the deep learning approaches outperformed those based on the physical model in terms of prediction accuracy, manifesting a better fitting of the vehicle trajectory due to the powerful representation learning ability. Another fact that can be seen from Table 1 is that the embedding of the interaction model significantly improved the prediction accuracy on both the NGSIM and HighD datasets. Additionally, the accuracy values obtained by S-LSTM, CS-LSTM, and P-LSTM had only slight differences. This implies that the interaction modeling through the spatial grid and based on the attention mechanism can both effectively express the mutual relationship. Adding any of these interactive models can improve the vehicle trajectory prediction accuracy.

#### 4.2.2. Exploration on Interaction Embedding

To evaluate the performance of interaction model embedding, three different methods were explored in this paper, i.e., embedding only at the current frame, embedding at each frame, and embedding with spatial–temporal coupling. We selected the pooling module as the interaction model of the network. To simplify the experiment, here, we output only one track for each target vehicle. The experimental results on the NGSIM dataset along with the runtime performance are shown in Table 2.

As can be seen, the prediction results obtained by the three interaction model embeddings were almost identical, with no obvious difference in the prediction accuracy. It can be reasoned that the interaction between vehicles is preserved for a certain time (even in the high-speed scenario). Hence, it is sufficient to consider the interaction at the current frame, while embedding at each frame duplicates the information. From the perspective of computational cost, the first embedding only occurs once, while the last two need to recalculate the interaction at each frame. Thus, the processing speed of the first embedding method is significantly faster than that of the other embedding methods, reaching about 98 Hz, indicating that such an embedding can effectively reduce the calculation amount of the model.

Therefore, as a trade-off between the prediction accuracy and the computation efficiency, the pooling module with the first way of embedding, i.e., was P-LSTM, was employed in the proposed network in the subsequent experiments.

### 4.3. Evaluation of Lane Orientation Information Integration

The qualitative and quantitative evaluations of the proposed prediction framework by integrating lane orientation information are reported as below.

#### 4.3.1. Qualitative Analysis

**Figure 4:**
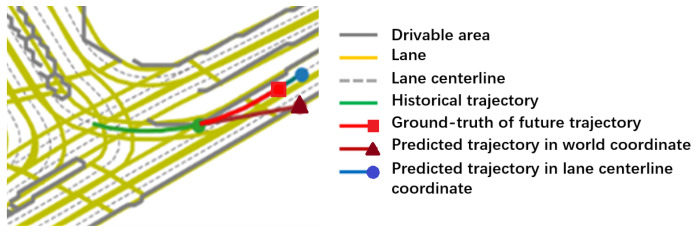
Visualization of the trajectories directly predicted in the world coordinate and by the proposed framework (which can be better viewed in color).

In the proposed prediction framework, the vehicle trajectory was transformed from the world coordinate to the lane centerline coordinate to encode the lane orientation information. To verify the efficacy of such an approach, an ablation study was conducted on the Argoverse benchmark, in which the road map is available. For qualitative analysis, a typical test scenario is firstly presented in Figure 4, with the visualization of the trajectories directly predicted in the world coordinate and by the proposed framework.

Obviously, the predicted trajectories in the world coordinate do not take into account the impact of road information. Thereby, the prediction result may conflict with the road structure, leading to unreasonable or even completely false trajectories. In comparison, the proposed framework can effectively use the candidate lane centerline to guide the prediction of trajectories that conform to the lane orientation.

#### 4.3.2. Quantitative Analysis

The quantitative evaluation results are shown in Table 3. In the ablation study, prediction methods with the proposed integration of lane orientation information from the map are indicated by the suffix “-map”. In this experiment, we also conducted single-mode prediction. It can be seen that by employing the lane centerline coordinate transform, the prediction accuracy significantly improved for both the physical-model- and deep-learning-based methods. The reason for the improvement is that in urban scenes, most of the vehicles travel along corresponding lanes on the road structure. The proposed coordinate system transform effectively uses the candidate lane centerline to guide the prediction and ensures the rationality of the predicted trajectory in terms of the road orientation, so as to reduce prediction errors. Nevertheless, the prediction accuracy was further boosted by considering the interaction in the P-LSTM-map, which also proved that both interaction and road map information play important roles in achieving more accurate predicted trajectories.

In addition, the proposed framework was compared with two different methods of integrating road map information: the rasterized map [15] and VectorNet [6]. We directly report their test results according to their original papers. As reported in the table, the prediction results by transforming the trajectory coordinate system were better than that by the rasterized map and VectorNet. In fact, both of these methods implicitly use the road map by encoding it as a part of the prediction network input and yield one deterministic predicted trajectory. In contrast, the proposed approach does not directly feed the road map to the network. Instead, it uses the lane orientation information as a strong constraint in pre-processing the input trajectory.

### 4.4. Evaluation of Multi-Modal Prediction

#### 4.4.1. Qualitative Analysis

**Figure 5:**
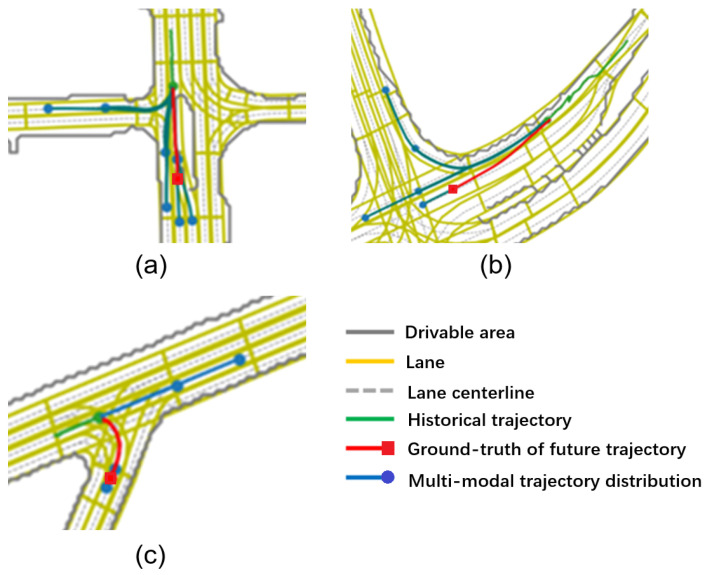
Visualization of multi-modal prediction results in different urban road scenarios (which can be better viewed in color). (**a**) Prediction at intersection. (**b**) Prediction at T-junction. (**c**) Prediction at merging.

In this section, the prediction framework is trained by the improved random multiple-choice learning, which considers both the interaction and lane orientation information. The experiments were conducted on the Argoverse dataset. Typical test scenarios with prediction results are firstly presented in Figure 5.

As visualized, the multi-modal vehicle trajectory prediction network integrated with interaction and road map information can dynamically generate future trajectories according to the change of urban road structures. Thus, it has a good generalization ability for urban scenes. Moreover, it takes into full account the characteristics of the dual-level modality of vehicle movement and is able to cover the motion mode of the ground-truth trajectory.

#### 4.4.2. Quantitative Analysis

The results of different prediction methods on the Argoverse dataset are compared in Table 4, with the indicator “- M” representing the multi-modal trajectory prediction based on the improved dual-level random multiple-choice learning. For a better comparison, the criteria of the minADE and minFDE were selected in this experiment.

It is obvious that multi-modal trajectory prediction methods such as LSTM-M-map and P-LSTM-M-map perform better than those only having single-mode predictions. Although LSTM-map and P-LSTM map can make reasonable predictions by transforming the vehicle trajectory into the coordinate system of the candidate lane centerline, they still have the following defects: On the one hand, the prediction network has difficulty learning the characteristics of the vehicles driving along different types of lane centerlines. For instance, the centerline of a turning lane and a straight lane should lead to different vehicle trajectories. On the other hand, even if driving along the same lane centerline, vehicles, according to their intention, may make different motions. In the proposed approach, the intention modes and motion modes are distinguished by candidate lane centerline feature vectors and pre-defined motion encodings. For the above reasons, the prediction network can generate a dual-level multi-modal trajectory distribution that can well cover the future motion of target vehicle, thus achieving more accurate prediction results.

Finally, the proposed framework P-LSTM-M-map was compared with another non-hierarchical multi-modal trajectory prediction method, i.e., the MFP-k [30], which simultaneously predicts six possible trajectories by an EM algorithm. For a fair comparison, another version of the proposed approach, called P-LSTM-map-k, was included in this experiment. This version of the approach learns to predict multi-modal trajectories through a generative network, which yields k latent variables, each conforming to a Gaussian distribution. Thus, the predicted modalities are non-hierarchical. Finally, inspired by LaneRCNN [31], we used the LaneRoI Encoder module instead of LSTM to encode the past motion and the local map topology. The LaneRoI Interactor module was used as the interaction model. We named it LaneRCNN-M and compared its performance with the original version and the recent work HOME [32], WIMP [33], and SceneTransformer [34]. The evaluation results on the Argoverse dataset are shown in Table 5. Note that the minFDE value of the MFP-k was not available in its original work, thus not reported here.

As reported in the prediction results, the multi-modal trajectory prediction method based on the improved dual-level random multiple-choice learning performs better than the version based on the generative network. The reason is that the learning of the generative network is easily biased, which leads to repeated sampling of modes with high probabilities, while modes with lower probabilities are fewer or even not sampled. In this way, the predicted trajectory distribution cannot span the entire probability space of the ground-truth. Furthermore, the MFP-k achieved the same accuracy as P-LSTM-map-k, yet still being inferior to the proposed P-LSTM-M-map. It can be assumed that the division of the trajectory modality into two levels facilitates the entire network learning, thus more effective than the utilization of the EM algorithm. Compared to LaneRCNN, the minFDE and minADE of LaneRCNN-M improved by 0.04 m and 0.1 m, respectively, further demonstrating the effectiveness and generalization ability of our two-stage random multiple-choice learning. Moreover, the improved approach also surpassed the recent work HOME and WIMP. However, there was still a slight gap between LaneRCNN-M and the SceneTransformer. We attribute this to the factorized attention and cross-attention utilized in SceneTransformer, which are assumed more effective in exploiting the time, agent, and road information based on a complex transformer structure. Since LaneRCNN-M is just a preliminary implementation without further optimization, there is still room for improvement, e.g., with the inspiration of SceneTransformer.

Nevertheless, the computational amount of our proposed approach was about 0.056 GFLOPs, and the number of trainable parameters was about 0.71 M. It can achieve a runtime speed of about 50 Hz under the experimental settings in Section 4.2.2, which is sufficient for most real-time applications.

## 5. Conclusions and Future Work

In this paper, an improved vehicle trajectory prediction architecture was proposed, which is based on the fusion of the information of lane orientation and vehicle interaction and intention, and it is capable of dealing with both highway and urban scenes. To integrate lane orientation information, a coordinate system transform in terms of the lane centerline was introduced in the pre-processing of the input trajectories. The transform is lightweight in computation, compatible with interaction models, and effective in guiding predicted trajectories to conform to road structures. Additionally, an improved dual-level random multi-choice learning was proposed to assist the network in learning different modality levels of the vehicle trajectory, incorporating both interaction and lane orientation information. With studies on the highway benchmarks of NGSIM and HighD, the performance of three interaction models, as well as the embedding style were explored and guidance for their deployment was presented. Through extensive comparison experiments on the additional Argoverse dataset, the proposed multi-modal prediction framework was demonstrated also to be effective at vehicle trajectory prediction in urban scenes.

In future work, the proposed approach will be adapted to trajectory prediction for other traffic participants such as pedestrians, and the integration with more effective architectures such as the transformer will also be explored. Due to the limitations of the available public datasets, the experiments only focused on the trajectory prediction of human-driven vehicles. With the popularity of autonomous vehicles, human-driven and automated vehicles will share the road and the road infrastructure will also change. Then, how to model the interaction between mixed vehicles and efficiently use the map information will also face new challenges and opportunities, which will be explored in our future work.

## Figures and Tables

**Table 1:** Vehicle trajectory prediction with various interaction models on the NGSIM and HighD datasets.

Dataset	NGSIM		HighD	
**Methods**	**ADE (m)**↓	**FDE (m)**↓	**ADE (m)**↓	**FDE (m)**↓
CV	3.42	6.68	1.49	2.83
IDM	3.40	6.60	1.52	2.86
LSTM (baseline)	3.19	6.27	1.43	2.76
S-LSTM	2.24	4.18	1.30	2.55
CS-LSTM	2.20	4.11	1.28	2.54
P-LSTM	2.21	4.17	1.25	2.52

**Table 2:** Prediction with different embedding methods on the NGSIM dataset.

Embedding Style	ADE (m) ↓	FDE (m) ↓	Runtime (Hz) ↑
Current frame	2.21	4.17	98
Each frame	2.21	4.15	12
Space–time coupling	2.20	4.14	8

**Table 3:** Ablation study of prediction approaches integrating lane orientation information on the Argoverse dataset. Test results of rasterized map and VectorNet are according to their original papers.

Methods	ADE (m) ↓	FDE (m) ↓
CV	3.95	8.56
CV-map	3.72	7.19
LSTM (baseline)	2.77	5.67
LSTM-map	1.67	3.58
P-LSTM-map	1.58	3.37
VectorNet	1.66	3.67
Rasterized Map	1.60	3.64

**Table 4:** Comparison of multi-modal vehicle trajectory prediction methods on Argoverse dataset.

Methods	minADE (m) ↓	minFDE (m) ↓
CV	3.95	8.56
CV-map	3.72	7.19
LSTM (baseline)	2.53	5.04
LSTM-map	1.67	3.58
P-LSTM-map	1.58	3.37
LSTM-M-map	0.88	1.74
P-LSTM-M-map	0.85	1.66

**Table 5:** Comparison of proposed framework and non-hierarchical multi-modal prediction methods on Argoverse dataset.

Methods	minADE (m) ↓	minFDE (m) ↓
P-LSTM-M-map	0.85	1.66
MFP-k	1.40	-
P-LSTM-map-k	1.40	3.15
LaneRCNN	0.90	1.45
LaneRCNN-M	0.86	1.35
WIMP	0.90	1.42
HOME	0.94	1.45
SceneTransformer	0.80	1.23

## Data Availability

Publicly available datasets were analyzed in this study. These data can be found here: https://data.transportation.gov/Automobiles/Next-Generation-Simulation-NGSIM-Vehicle-Trajector/8ect-6jqj, https://www.highd-dataset.com/, and https://www.argoverse.org/ (all accessed on 4 June 2022).

## Abbreviations

The following abbreviations are used in this manuscript:

LSTM	Long Short-Term Memory
NN	Neural Network
CNN	Convolutional Neural Network
GNN	Graph Neural Network
GAN	Generative Adversarial Network
FC	Fully Connected
